# Identification of Target Proteins Involved in Cochlear Hair Cell Progenitor Cytotoxicity following Gentamicin Exposure

**DOI:** 10.3390/jcm11144072

**Published:** 2022-07-14

**Authors:** Camron Davies, Rahul Mittal, Crystal Y. Li, Hannah Marwede, Jenna Bergman, Nia Hilton, Jeenu Mittal, Sanjoy K. Bhattacharya, Adrien A. Eshraghi

**Affiliations:** 1 Department of Otolaryngology, Hearing Research and Cochlear Implant Laboratory, University of Miami Miller School of Medicine, Miami, FL 33136, USA; cdavi191@med.fiu.edu (C.D.); r.mittal11@med.miami.edu (R.M.); cyl14@med.miami.edu (C.Y.L.); hxm352@med.miami.edu (H.M.); jberg062@med.fiu.edu (J.B.); niahilton2022@gmail.com (N.H.); j.mittal@med.miami.edu (J.M.); 2 Herbert Wertheim College of Medicine, Florida International University, Miami, FL 33199, USA; 3 Miami Integrative Metabolomics Research Center, Bascom Palmer Eye Institute, University of Miami Miller School of Medicine, Miami, FL 33136, USA; sbhattacharya@med.miami.edu; 4 Department of Neurological Surgery, University of Miami Miller School of Medicine, Miami, FL 33136, USA; 5 Department of Biomedical Engineering, University of Miami, Coral Gables, FL 33146, USA; 6 Department of Pediatrics, University of Miami Miller School of Medicine, Miami, FL 33136, USA

**Keywords:** HEI-OC1, organ of Corti, gentamicin ototoxicity, mass spectrometry, high-performance liquid chromatography, confocal microscopy

## Abstract

Given the non-labile, terminal differentiation of inner-ear sensory cells, preserving their function is critical since sensory cell damage results in irreversible hearing loss. Gentamicin-induced cytotoxicity is one of the major causes of sensory cell damage and consequent sensorineural hearing loss. However, the precise molecular mechanisms and target proteins involved in ototoxicity are still unknown. The objective of the present study was to identify target proteins involved in gentamicin-induced cytotoxicity to better characterize the molecular pathways involved in sensory cell damage following ototoxic drug administration using House Ear Institute-Organ of Corti 1 (HEI-OC1) cells and high-performance liquid chromatography coupled with tandem mass spectrometry (HPLC-MS/MS). We identified several unique proteins involved in gentamicin-induced cytotoxicity, expression of which were further confirmed using confocal microscopy. Further investigation of these pathways can inform the design and discovery of novel treatment modalities to prevent sensory cell damage and preserve their function.

## 1. Introduction

Hearing loss is one of the most common neurosensory disorders affecting humans [1,2]. In the United States alone, more than 48 million individuals over 12 years old, approximately 20% of the population, are affected by some type of hearing loss (HL) [3]. Moreover, the prevalence of HL continues to increase with each decade of life. Approximately 25% of adults between 65 and 74 years old and 50% of adults older than 75 report disabling HL. These figures make HL one of the top four chronic health conditions affecting older adults. Given the widespread prevalence of HL and its profound impact on an individual’s physical, psychological, and social well-being, identifying and understanding the contributing factors to HL can have a profound personal and societal impact [4,5,6].

One common yet poorly understood cause of sensorineural HL is the ototoxic effects of frequently prescribed medications. Based on data from the National Health and Nutrition Examination Survey (NHANES), 25% of adults between 20 and 69 years old used ototoxic medications such as NSAIDs, antineoplastic drugs, loop diuretics, and aminoglycoside antibiotics [7]. A study showed that the prevalence of potentially ototoxic medication use reached 91% among older adults [8]. Moreover, while drug-induced ototoxicity for many drugs often resolves after their discontinuation, the use of platinum-derived chemotherapy and aminoglycosides can lead to permanent HL [9,10]. The present study specifically seeks to characterize the proteins involved in the apoptotic pathway of one such aminoglycoside, gentamicin.

Aminoglycosides are a potent broad-spectrum antibiotic with particularly effective activity against Gram-negative bacteria, including *Escherichia coli*, *Klebsiella pneumoniae*, and *Enterobacter cloacae* [11,12]. Given its high efficacy and low cost, gentamicin is one of the most commonly used aminoglycosides against severe infections such as sepsis and endocarditis [13,14]. Despite these advantages, aminoglycosides such as gentamicin have a notoriously narrow therapeutic index due to their dose-dependent nephrotoxic and ototoxic effects [5,15]. Specifically, gentamicin ototoxicity has been found to occur in as many as 20% of patients undergoing treatment over multiple days and is characterized by both vestibular and hearing dysfunction [5,16].

Aminoglycosides are believed to induce ototoxicity through the generation of reactive oxygen species (ROS), which subsequently cause widespread cellular damage and thereby induce apoptosis in cochlear hair cells (HCs) [17,18]. Interestingly, this mechanism is hypothesized to be shared by many causes of sensorineural hearing loss (SNHL), including noise-induced hearing loss and other ototoxic drugs such as cisplatin and acetaminophen [19]. Since cochlear HCs cannot regenerate, their apoptosis causes permanent SNHL. While several studies have identified and hypothesized these mechanisms for hearing loss [20,21,22], the precise molecular apoptotic pathways remain unclear.

Much of the current literature examining drug-induced ototoxicity focuses on the mediators of cellular damage and the apoptotic pathways to identify mechanisms to reduce cellular damage or inhibit the key apoptotic steps [23]. For example, the production and effect of reactive oxygen species in cellular damage, the subsequent activation of caspase pathways, and the role of calcium channels have been well-described. However, there is increasing evidence that aminoglycoside ototoxicity is regulated by processes before extensive cellular damage is present and before activation of main apoptotic pathways [24,25]. There is a need to identify novel targets involved in gentamicin-induced cytotoxicity that will pave the way to developing effective therapeutic strategies.

Given the prevalence and impact of HL and the role of ototoxic drugs such as gentamicin, identifying the critical mediators of sensory cell death presents a unique opportunity for intervention along their apoptotic pathway. Identifying downstream apoptotic mediators may help develop effective treatment modalities to reduce HC death. Among the techniques used to identify and characterize host pathways, tandem mass spectrometry (MS/MS or MS) has recently become a powerful tool [26,27]. MS has proven increasingly valuable for cellular research due to its exceptional ability to characterize molecular pathways in detail by obtaining a significant amount of quantitative and qualitative data on biological substances [28,29]. In the present study, MS was chosen to identify potential target proteins following gentamicin exposure given its high sensitivity, high accuracy, and broad analytical potential. Additionally, HEI-OC1 cells were selected as a model for HCs as they represent a progenitor for both HCs and the supporting cells of the organ of Corti, expressing cochlear HC markers such as prestin, Myo7a, ATOH1, BDNF, calmodulin, and calbindin [30]. Identifying critical cellular mediators of HC damage will allow the development of more effective otoprotective therapies for gentamicin ototoxicity and even other causes of HL.

## 2. Materials and Methods

### 2.1. Cell Culture

HEI-OC1 cells (kindly provided by Dr. Federico Kalinec, UCLA) were cultured under permissive conditions (33 °C, 10% CO_2_) in high-glucose Dulbecco’s Eagle’s medium (DMEM) containing 10% fetal bovine serum (FBS) without antibiotics, as described in previous studies [31,32,33,34].

### 2.2. Gentamicin Treatment

**Figure 1:**
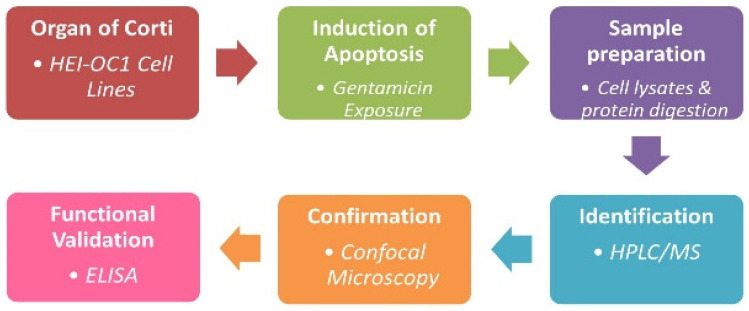
Schematic representation of experimental design for HPLC-MS/MS.

HEI-OC1 cells were treated with 1 mM and 3 mM concentrations of gentamicin for 8 h and 24 h. In this study, the concentrations of 1 mM and 3 mM gentamicin used were based on previous studies [35,36]. After incubation, cells were washed, and lysates were prepared, followed by the determination of protein concentration, as described in previous studies. A schematic representation of the experimental design is shown in Figure 1.

### 2.3. Sample Denaturation/Reduction/Alkylation

To denature, 15 µL of 10 M urea in 50 mM ammonium bicarbonate was added to the samples. Then, 2 µL of 125 mM DTT in 50 mM ammonium bicarbonate was added to reduce the proteins. Samples were then incubated at room temperature for 1 h. After incubation, 5 µL of 90 mM Iodoacetamide in 50 mM ammonium bicarbonate was added to each of the samples for alkylation. Samples were then incubated in the dark and at room temperature for 30 min. Next, 3.33 µL of 125 mM DTT in 50 mM ammonium bicarbonate was added to the samples for iodoacetamide quenching. Samples were then incubated in the dark and at room temperature for 1 h. Following this, to dilute the urea to 1 M, 116.67 µL of 50 mM ammonium bicarbonate was added, leaving the samples at a volume of 150 µL.

### 2.4. Sample Enzymatic Digestion

For digestion, a trypsin stock solution of 0.1 µg/µL was prepared. Then, 3.33 µL of the stock solution was added to each sample. The samples were incubated overnight at 37 °C. After incubation, 50% formic acid (FA) was added to each sample. Samples were then stored at −20 °C until they were ready for MS.

### 2.5. Desalting and Protein Enrichment

Before the proteins were run through MS, they were desalted using Pierce C18 Spin Tips. Desalting is critical because salt and urea in the sample can interfere with MS ionization. TFA solutions were then prepared (2.5% TFA, 0.1% TFA in 80% ACN, 0.1% TFA, and 0.1% TFA in 5% ACN). To adjust the overall TFA concentration to 0.5%, 40.6 µL of 2.5% TFA was added to each sample. C18 spin tips were then inserted into the spin adapter that was seated in a microcentrifuge tube. Next, 20 µL of 0.1% TFA in 80% ACN was added to the C18 tip to wet it and then centrifuged at 1000× *g* for 1 min. Next, to equilibrate the tip, 20 µL of 0.1% TFA was added to the tip; then, it was centrifuged at 1000× *g* for 1 min. These steps prepared the column to bind to the proteins in the following steps. The C18 spin tip and adapter were then transferred to a new microcentrifuge tube. Briefly, 50 µL of one of the samples was added to the C18 tip and centrifuged at 1000× *g* for one minute. In addition, a 50 µL volume of the sample was added, and the centrifugation was repeated until all of the samples had run through the tip. Then, the tip was washed with 20 µL 0.1% TFA, followed by a 1 min centrifuge at 1000× *g*, repeated one additional time. By this point, the salts should have eluted, and the majority of the proteins should have been bound inside the column. Then, the C18 spin tip and adapter were transferred to a new microcentrifuge tube. The sample was eluted by adding 20 µL of 0.1% TFA in 80% ACN and was centrifuged at 1000× *g* for 1 min. This step was repeated once more. This process was conducted for all the samples. At this point, the desalted protein samples were collected, speed-vacuumed to near dryness, and then resuspended in 50 µL of 0.1% FA, 2% ACN, in MilliQ for MS analysis. Samples were aliquoted in a mass spec tube and run on a Q Exactive Hybrid Quadrupole Orbitrap Mass Spectrometer.

### 2.6. Data Analysis

The data analysis was performed using Proteome discoverer (ThermoFisher scientific, Waltham, MA, USA) and MetaboAnalyst (https://www.metaboanalyst.ca/MetaboAnalyst/upload/StatUploadView.xhtml, accessed on 1 December 2020) software programs, with parameters as described in detail in previous studies [37,38,39].

### 2.7. Confocal Microscopy

For immunofluorescence, HEI-OC1 cells were cultured in 8-well chamber slides and treated with 1 mM and 3 mM concentrations of gentamicin for 8 h and 24 h. After incubation, cells were washed three times with PBS buffer, followed by fixation and permeabilization with BD cytofix and cytoperm reagent (BD Biosciences, San Jose, CA, USA) for 30 min. After washing, the cells were blocked with 3% normal goat serum (NGS) for 20 min and then incubated with either anti-Sap30bp antibody, TAO1 kinase antibody, or cleaved caspase 3 antibody (Abcam, Cambridge, MA, USA) overnight at 4 °C, followed by washing and incubation with Alexa Fluor 568 secondary antibody (Life Technologies, Carlsbad, CA, USA) for 90 min at room temperature. After washing, samples were mounted in an antifade Vectashield solution containing 4, 6-diamidino-2-phenylindole (DAPI) (Vector Laboratories, Burlingame, CA, USA). The cells were viewed with a Zeiss LSM 710 microscope (Carl Zeiss, Germany), and images were assembled using Adobe photoshop 7.0.

To determine mean signal intensity, the mean green signal intensity was measured as the average of 10 regions of interest (ROI) and normalized using the mean signal background intensity. The size and location of each ROI were consistent for all images. The mean signal intensity was measured and calculated using ImageJ version 1.52 k software (Bethesda, MD, USA) [40,41,42].

### 2.8. Cleaved Caspase 3 Determination

The levels of cleaved caspase 3 were quantitatively determined in HEI-OC1 cell homogenates using an ELISA kit as per the manufacturer’s instructions (Abcam, Cambridge, MA, USA).

### 2.9. Statistical Analysis

Two-tailed Student’s *t*-test was used to establish significant differences between groups. *p* values of less than 0.05 were considered significant. All statistical analyses were performed using SPSS software version 28 (IBM, Armonk, NY, USA).

## 3. Results

### 3.1. Upregulated Proteins in Response to Gentamicin Ototoxicity in HEI-OC1 Cells

We observed that several host proteins were upregulated following treatment with gentamicin, compared with untreated control samples. We identified various novel target proteins, as summarized in Table 1, such as Sap30-binding protein, serine/threonine-protein kinase TAO1, myocyte-specific enhancer factor 2D, Bcl-2 homologous antagonist/killer, caspase-9 (Fragment), targeting protein for Xklp2, microtubule-associated protein 1S, MKIAA0655 protein (Fragment), growth arrest-specific protein 2 (GAS-2), DNA-directed RNA polymerase II subunit, nucleolysin TIA-1, caspase-4 (CASP-4), and FAS-associated death domain protein. There were differences in the levels of upregulation of host proteins depending on the exposure time and concentration of gentamicin. The majority of upregulated proteins were regulators of apoptosis pathways.

### 3.2. Common Target Proteins Upregulated Following Treatment of HEI-OC1 with Two Doses of Gentamicin

**Figure 2:**
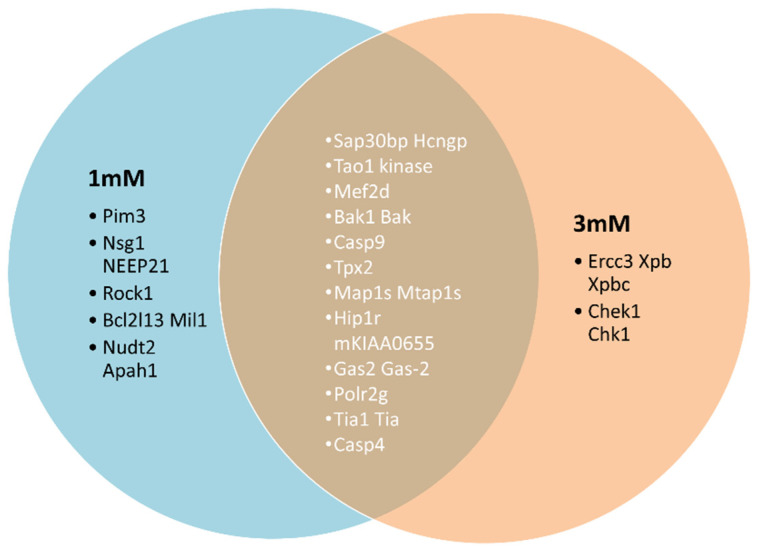
Venn diagram showing common overexpressed proteins following treatment of HEI-OC1 to 1 mM and 3 mM concentrations of gentamicin.

We selected top proteins upregulated at 1 mM and 3 mM doses of gentamicin. At 1 mM, we observed upregulation of serine/threonine-protein kinase pim-3, whereas enhanced levels of ERCC3 XBP protein were observed at 3 mM (Table 1). Interestingly, we observed that Sap30bp and TAO1 kinase were upregulated at both 1 mM and 3 mM concentrations of gentamicin, as shown in the Venn diagram in Figure 2.

### 3.3. Sap30bp Immunostaining in HEI-OC1 via Confocal Microscopy

**Figure 3:**
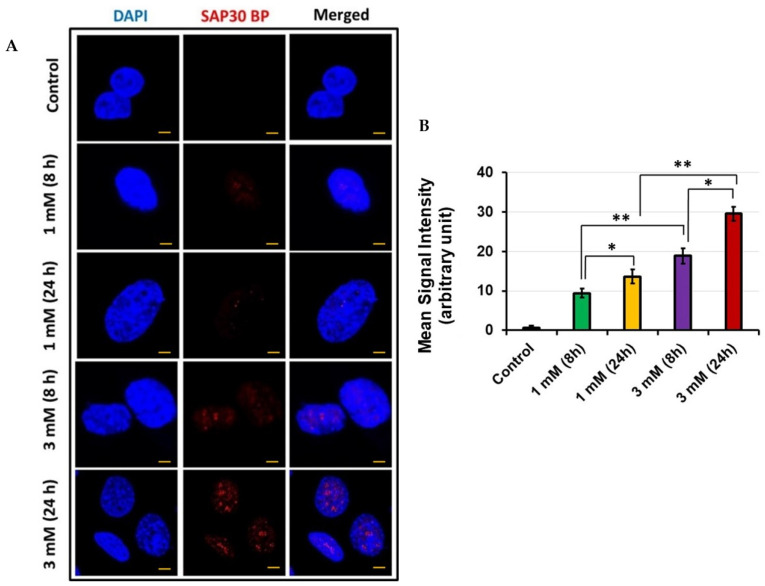
Sap30bp immunostaining: (**A**) representative photomicrographs showing Sap30bp expression in HEI-OC1 cells treated with gentamicin. Red: Sap30bp; Blue: cell nuclei. Scale Bars: 20 µM; (**B**) ImageJ software was used to determine mean signal intensity for Sap30bp. * *p* < 0.05 or ** *p* < 0.01.

To confirm the results of MS, we treated HEI-OC1 with 1 mM and 3 mM concentrations of gentamicin for 8 h and 24 h, stained with an anti-Sap30bp antibody, and subjected to confocal microscopy. In agreement with our MS results, we observed intense immunostaining of Sap30bp in HEI-OC1 exposed to 3 mM concentration compared with 1 mM gentamicin for 24 h (Figure 3A). The mean signal intensity for Sap30bp immunostaining was significantly higher in samples subjected to 3 mM gentamicin treatment for 24 h compared with cells treated for 8 h (*p* < 0.05). In addition, the mean signal intensity for Sap30bp immunostaining was significantly higher in HEI-OC1 treated with 3 mM compared with cells exposed to 1 mM gentamicin concentration for 24 h (*p* < 0.01) (Figure 3B).

### 3.4. TAO1 Kinase Expression in HEI-OC1 via Confocal Microscopy

**Figure 4:**
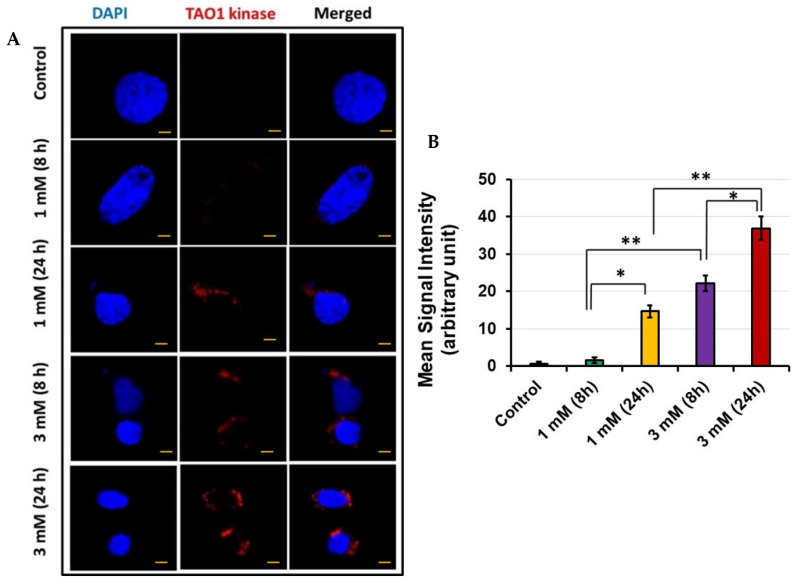
TAO1 kinase immunostaining: (**A**) representative photomicrographs showing Sap30bp expression in HEI-OC1 cells treated with gentamicin. Red: TAO1 kinase; Blue: cell nuclei. Scale Bars: 20 µM; (**B**) ImageJ software was used to determine mean signal intensity for TAO1 kinase immunostaining. * *p* < 0.05 or ** *p* < 0.01.

Since we also observed increased levels of TAO1 kinase using MS, we confirmed its expression with confocal microscopy following exposure of HEI-OC1 to 1 mM and 3 mM gentamicin concentrations for 8 and 24 h. At 1 mM gentamicin concentration, there was no TAO1 kinase expression at 8 h, whereas increased expression was observed at 24 h (Figure 4A). On the other hand, when the cells were exposed to 3 mM gentamicin, there was TAO1 kinase expression at 8 h that further increased at 24 h. As observed with Sap30bp immunostaining, the mean signal intensity for TAO1 kinase immunostaining was significantly higher in samples subjected to 3 mM gentamicin treatment for 24 h than in cells treated for 8 h (*p* < 0.05). In addition, the mean signal intensity for TAO1 kinase immunostaining was significantly higher in HEI-OC1 treated with 3 mM than in cells exposed to 1 mM gentamicin concentration for 24 h (*p* < 0.01) (Figure 4B).

### 3.5. Cleaved Caspase 3 Determination

**Figure 5:**
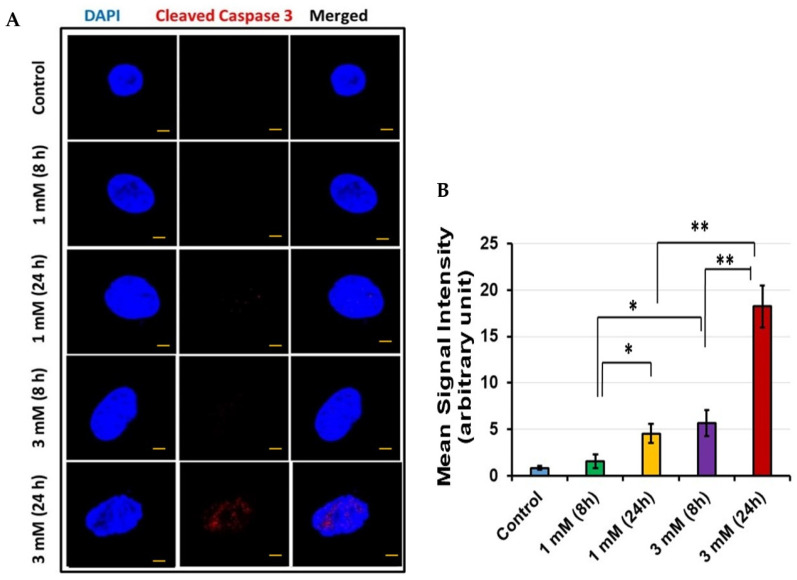
Cleaved caspase 3 immunostaining: (**A**) Representative photomicrographs showing cleaved caspase 3 expression levels in HEI-OC1 cells treated with gentamicin. Red: cleaved caspase 3; Blue: cell nuclei. Scale Bars: 20 µM; (**B**) ImageJ software was used to determine mean signal intensity for cleaved caspase 3 immunostaining. * *p* < 0.05 or ** *p* < 0.01.

**Figure 6:**
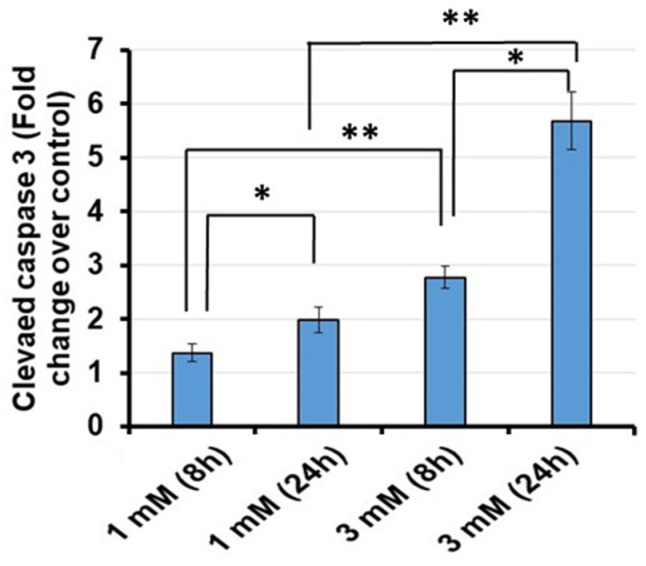
Cleaved caspase 3 levels in HEI-OC1 cell homogenates exposed to gentamicin were determined using an ELISA kit. * *p* < 0.05 or ** *p* < 0.01.

To confirm that apoptosis was occurring in conjunction with increased expression of Sap30bp and TAO1 kinase, cleaved caspase 3 was assayed in HEI-OC1 cells via confocal microscopy. As a convergent point for both the intrinsic and extrinsic apoptotic pathways, cleaved caspase 3 is an ideal marker for cellular apoptosis. With increasing exposure and concentration of gentamicin, elevated cleaved caspase 3 expression levels were observed in HEI-OC1 cells (Figure 5A). In addition, the mean signal intensity for cleaved caspase 3 immunostaining was significantly higher in HEI-OC1 treated with 3 mM than in cells exposed to 1 mM gentamicin concentration for 24 h (*p* < 0.01) (Figure 5B). To further confirm the results of confocal microscopy, we quantitatively determined cleaved caspase 3 levels using an ELISA kit. In agreement with our confocal microscopy data, quantification of cleaved caspase 3 levels via ELISA confirmed that its enhanced expression coincides with increasing exposure to gentamicin (Figure 6).

## 4. Discussion

In this study, we identified novel target proteins involved in gentamicin-induced cytotoxicity using tandem mass spectrometry (MS/MS, referred to as MS from here on). We observed significant upregulation of TAO1 kinase in HEI-OC1, especially after treatment with high doses of gentamicin for 24 h. TAO-1 is a serine/threonine-protein kinase known to activate the MAPK cascade, regulating vital cellular processes such as mitosis, proliferation, differentiation, and immune responses [43]. Within the cell cycle, previous studies demonstrate that TAO-1 can shorten the G1 phase and skip a transient G0-like state to accelerate cell cycle progression [44]. TAO-1 has also been shown to regulate apoptotic changes such as cell contraction, pyknosis, karyorrhexis, membrane blebbing, and apoptotic body formation via the MAPK8/JNK pathway [45,46].

It is important to note that under physiologic conditions, cochlear HCs are terminally differentiated and in a quiescent state. However, an analysis of transcriptomic changes in mouse cochlea after gentamicin administration found increased expression of genes involved in cell cycle progression, G2 phase, and G2/M phase, indicating a disruption in HC’s quiescent state. Interestingly these transcriptomic changes occurred before a significant stress response was seen and before the initiation of apoptosis [24]. Additionally, the JNK and NF–κB signaling pathways were implicated as early responders after gentamicin exposure, while genes involved in the initiation and execution of apoptosis were not significantly induced. These findings indicate that early responders within the cell cycle may be key regulators of aminoglycoside ototoxicity. This is supported by Karasawa et al., who identified HSP73 and calreticulin as key gentamicin binding proteins via pull-down assays. These regulatory proteins have essential functions in protein folding and are otoprotective against gentamicin [25,47]. Their loss of function after gentamicin binding further supports the instability of HC’s post-mitotic state as a key regulator of HC apoptosis. The results of the present study echo this hypothesis. In the absence of gentamicin, there was very low TAO-1 activity. However, after exposure to gentamicin, TAO-1 expression rapidly increased. These results, along with TAO-1′s known role in apoptosis, further implicate TAO1 kinase in gentamicin-induced cell damage.

In addition to TAO1 kinase, we observed significant upregulation of Sap30bp when HEI-OC1 cells were exposed to 300 µM gentamicin for 24 h. Sap30bp, also known as HTRG, HTRP, or HCNGP, encodes a transcriptional regulator protein that localizes to the nucleus where it interacts with Sap30, a component of the histone deacetylase complex (HDAC) and, therefore, is believed to repress transcription by promoting HDAC activity [48,49,50]. While it is relatively understudied, Sap30bp is ubiquitously present in a wide range of tissues and, in the current literature, has been associated with increased cell death, suggesting that it represses pro-survival pathways [51,52,53]. Thus, in the context of this study, and the existing literature implicating significant transcriptional changes in HC death, regulators such as Sap30-bp are particularly interesting targets. However, it is important to note that more research is needed to verify this role.

Given the implicated functions of both Sap30bp and TAO1 kinase, cleaved-caspase-3 was assayed as a marker of apoptosis. Caspase-3 is a well-validated cochlear HC apoptotic marker [54,55]. It is activated by initiator caspases such as caspase-8 or caspase-9 via the extrinsic and intrinsic pathways, respectively. Both pathways result in the release of cytochrome c from the mitochondria, which oligomerizes with other cytosolic factors to form a complex that cleaves procaspase 3 into active cleaved-caspase-3, which then propagates the apoptotic pathway [54,56]. Given its role as one of the late downstream effector enzymes for both the intrinsic and extrinsic apoptotic pathways, cleaved-caspase-3 is an ideal marker for apoptosis [54,57]. Indeed, in the present study, there was significant upregulation of cleaved-caspase-3 following gentamicin exposure, particularly after treatment with high doses of gentamicin for 24 h. These findings are supported by previous studies demonstrating increased caspase 3-like activity in TAO1 kinase transfected cells [45].

Notably, the cleaved-caspase-3 confocal microscopy images were visually less impressive than the images for TAO-1 and Sap30bp, only appearing to show the presence of cleaved-caspase-3 at the 3 mM 24 h dose/timepoint. This is likely the result of several factors related to cleaved-caspase-3. Given its role as a late downstream effector protease in the apoptotic pathway, cleaved-caspase-3 is minimally expressed until late in the apoptotic process, only being visualized in conjunction with pyknotic nuclei [54]. This tends to result in an “all or nothing” visual effect. Additionally, there is significant variation in the onset of cleaved-caspase-3 activation between different experimental conditions, varying widely with different models and exposures. For example, in a study of chinchilla cochlea explants, confirmation of cleaved-caspase-3 expression was seen as soon as 6 h after noise exposure, while in a study of gentamicin-treated chick cochlea, cleaved-caspase-3 was not seen on confocal microscopy until at least 30 h post-exposure [54,57]. In the present study, despite the apparent lack of cleaved-caspase-3 on confocal microscopy, its presence and relative increase after gentamicin exposure was confirmed via ELISA, which can take a larger sample into account.

This study also demonstrated the importance of mass spectrometry. MS is the current technology of choice for detecting and quantifying proteins and metabolites, as it is adaptable to virtually any type of sample, ranging from cells and neurons to subcellular organelles, tissues, and even whole embryos [58,59,60,61,62,63,64]. Mass spectrometers can directly detect intact proteins, peptides, post-translational modifications, and metabolites with high specificity, usually sub-mDa (sub-ppm) mass accuracy, and the capability for both discovery (untargeted) and targeted studies. MS does not require functional probes, antibodies, or a priori knowledge of molecules produced in the system. Additionally, MS workflows can be highly reproducible (such as quantitative error <5% relative standard deviation), allowing for both absolute and relative quantification [65].

The advent of high-resolution MS has led to the discovery of novel, specifically expressed protein biomarkers that may be involved in the pathophysiology of diseases that are difficult to identify by immunohistochemistry and histologic morphology alone [66]. The importance of MS in identifying the target proteins involved in virtually any disease process makes it an invaluable discovery tool since MS can provide a catalog of novel target proteins implicated in health and disease.

In summary, our results showed that gentamicin exposure upregulates Sap30bp and TAO1 kinase in HEI-OC1 cells in a concentration and time-dependent manner. This enhanced expression of TAO-1 and Sap30bp mediates cell damage and cytotoxicity through the upregulation of cleaved caspase 3. While these in vitro results are promising, in vivo confirmation in animal models is a necessary next step to replicate the complex physiological environment of the mammalian cochlea and is desired for future drug screening. In future studies, it will be worthwhile to investigate the efficacy of pharmacological inhibitors or RNAi-based approaches to block the functions of these proteins, as has been demonstrated with other targets [67,68,69,70,71,72,73].

## Figures and Tables

**Table 1:** A summary of upregulated target genes and corresponding protein profiles in HEI-OC1 exposed to 1 mM and 3 mM concentrations of gentamicin. Red, yellow, and green colors denote high, intermediate, and low expression profiles.

Gene Names	1 mM	3 mM	Protein Names
*Sap30bp Hcngp*	27	38	SAP30-binding protein
*Taok1*	24	37	TAO1 kinase
*Mef2d*	20	37	Myocyte-specific enhancer factor 2D
*Bak1 Bak*	35	35	Bcl-2 homologous antagonist/killer
*Casp9*	27	34	Caspase-9 (Fragment)
*Tpx2*	33	32	Targeting protein for Xklp2
*Map1s Mtap1s*	20	26	Microtubule-associated protein 1S
*Hip1r mKIAA0655*	16	26	MKIAA0655 protein (Fragment)
*Gas2 Gas-2*	28	24	Growth arrest-specific protein 2 (GAS-2)
*Polr2g*	18	23	DNA-directed RNA polymerase II subunit
*Tia1 Tia*	11	21	Nucleolysin TIA-1
*Casp4 Casp11 Caspl Ich3*	24	21	Caspase-4 (CASP-4)
*Fadd Mort1*	11	20	FAS-associated death domain protein
*Bik Biklk*	28	18	Bik protein
*Ddit3 Chop Chop10 Gadd153*	18	17	DNA damage-inducible transcript 3 protein
*Csnk2a1 Ckiia*	11	16	Casein kinase II subunit alpha (CK II alpha)
*Dap3*	15	16	28S ribosomal protein S29, mitochondrial
*Epo*	20	15	Erythropoietin
*Cyfip2 Kiaa1168 Pir121*	14	15	Cytoplasmic FMR1-interacting protein 2
*Pim3*	13	13	Serine/threonine-protein kinase pim-3
*Nsg1 NEEP21*	11	12	Neuronal vesicle trafficking protein 1
*Birc6*	7	12	UBIQUITIN_CONJUGAT_2 domain protein
*Hip1r*	8	11	Huntingtin-interacting protein 1
*Rock1*	12	9	Rho-associated protein kinase 1
*Fas*	7	9	Fas
*Bcl2l13 Mil1*	18	8	Bcl-2-like protein 13 (Bcl2-L-13)
*Cckbr*	10	8	Gastrin/cholecystokinin type B receptor

## Data Availability

All the data presented in this study are available within the article.
